# Miniaturized Pathogen Detection System Using Magnetic Nanoparticles and Microfluidics Technology

**DOI:** 10.3390/mi15101272

**Published:** 2024-10-20

**Authors:** Benjamin Garlan, Amine Rabehi, Kieu Ngo, Sophie Neveu, Reza Askari Moghadam, Hamid Kokabi

**Affiliations:** 1 Group of Electrical Engineering of Paris (GeePs), Sorbonne Université, CNRS UMR8507, 75005 Paris, France; benjamin.garlan@gmail.com (B.G.); amine.rabehi@gmail.com (A.R.); hamid.kokabi@sorbonne-universite.fr (H.K.); 2 Laboratoire de Réactivité de Surface, LRS, Sorbonne Université, UMR CNRS 7197, 75252 Paris, France; kieu.ngo@sorbonne-universite.fr; 3 Laboratoire Physicochimie des Electrolytes et Nanosystèmes InterfaciauX, PHENIX, Sorbonne Université, UMR CNRS 8234, 75252 Paris, France; sophie.neveu@sorbonne-universite.fr; 4 CNRS, INSERM, Laboratoire d’Imagerie Biomédicale, LIB, Sorbonne Université, 75006 Paris, France

**Keywords:** microfluidics, pathogen detection, magnetic nanoparticle, frequency mixing method

## Abstract

Rapid detection of a biological agent is essential to anticipate a threat to the protection of biodiversity and ecosystems. Our goal is to miniaturize a magnetic pathogen detection system in order to fabricate an efficient and portable system. The detection device is based on flat, multilayer coils associated with microfluidic structures to detect magnetic nanoparticles linked to pathogen agents. One type of immunological diagnosis is based on the measurement of the magnetic sensitivity of magnetic nanoparticles (MNPs), which are markers connected to pathogens. This method of analysis involves the coupling of antibodies or antigen proteins with MNPs. Among the available magnetic techniques, the frequency mixing method has a definite advantage by making it possible to quantify MNPs. An external magnetic field composed of a low- and a high-frequency field is applied to the sample reservoir. Then, the response signal is measured and analyzed. In this paper, magnetic microcoils are implemented on a multilayer Printed Circuit Board (PCB), and a microfluidics microstructure is designed in connection with the planar coils. Simulation software, COMSOL version 5.3, provides an analytical perspective to choose the number of turns in magnetic coils and to understand the effects of changing the shape and dimensions of the microfluidics microstructure.

## 1. Introduction

Recent advances in magnetic microfluidic biosensors have revolutionized the field by integrating magnetic technology with microfluidic systems for enhanced biosensing capabilities. These biosensors utilize the unique properties of magnetic nanoparticles, such as their manipulation by external magnetic fields, for various applications in health care, environmental monitoring, and food safety. Key advancements include the development of miniaturized systems for point-of-care testing, high sensitivity and specificity achieved through functionalization of magnetic nanoparticles with biomolecules, and the integration of microfluidic channels for precise sample manipulation. These biosensors offer rapid and cost-effective detection of biomolecules, pathogens, and toxins, making them promising tools for a vast range of applications and ideal for point-of-care diagnostics. Additionally, their compatibility with standard silicon-integrated circuit technology enables their integration into handheld, portable devices. Magnetic biosensors show promise for the revolutionization of disease diagnosis and biological research methods, particularly in resource-limited settings [1,2,3,4].

The new focus on portable biosensing devices highlights the importance of integrating magnetic technology with microfluidic systems, particularly through the mixing frequency technique. This technique allows for the rapid, sensitive, and specific detection of biomolecules, pathogens, and cells. By employing magnetic markers manipulated by external or integrated magnetic fields within microfluidic channels, researchers are developing compact and reliable biosensors [5].

MNPs, particularly those in the size range of 1–100 nm, show unique physical, magnetic, and chemical properties due to their small size, making them suitable for biosensing applications. The synthesis of MNPs involves various methods, including physical, chemical, and microbial approaches, with surface coating being crucial for stabilization and functionalization. MNPs demonstrate superparamagnetism, making them ideal for manipulation and detection in sensing devices. Electrochemical sensing strategies utilizing MNPs offer enhanced sensitivity and low limits of detection, with MNPs acting as labels or immobilization supports on electrode surfaces. Examples include the use of core-shell MNPs for the detection of analytes such as organophosphorus pesticides and ochratoxin-A [6]. In another work, a microfluidic platform integrated with micro-fluxgate and microcoil technologies was proposed for to trap and detect magnetic beads. The micro-spiral coil traps the beads, while the micro-fluxgate detects the induced weak magnetic field [7]. In another paper, a highly sensitive method was presented for the detection of aflatoxin B1 in food and crops using frequency mixing magnetic detection technology. The detection method demonstrates a limit of detection comparable to that of the conventional competitive ELISA reference method, making it a promising approach for on-site testing [8].

In another study, a rapid detection system was developed for cholera toxin B detection using the magnetic frequency mixing technique [9]. A novel assay was also proposed for the detection and quantification of penicillin and kanamycin residues in whole-fat milk using magnetic immunodetection technology. This portable and sensitive method involves coating immunofiltration columns with penicillin G and kanamycin conjugates, then pre-incubating biotinylated antibodies with antibiotic-containing samples. The bound antibodies are labeled with streptavidin-coated magnetic nanoparticles and quantified by a frequency mixing magnetic detection technique [10].

There are more examples of the detection of pathogens or biomarkers using magnetic mixing techniques, including for the detection of SARS-CoV-2, Thyroid Stimulating Hormone (TSH), and C-Reactive Protein (CRP) [11,12,13]. The experimental results show 97% sensitivity and 92% specificity for SARS-CoV-2 detection based on tests applied on 170 patients. Details of serological magnetic detection of SARS-CoV-2 antibodies in human serum and the preparation procedure of penicillin Bovine Serum Albumin (BSA) and kanamycin–BSA conjugate can be found in related references [10,11]. A significant biomarker for the detection of inflammation is CRP protein, which can be found in the blood of patients. One proposed method for CRP detection is the magnetic mixed-frequency approach. The details of chemical and biomaterial specifications, MNP characteristics, and the procedure of immobilization of biomolecules have been explained in the literature [13].

This paper presents a novel prototype of a miniaturized magnetic detection device designed to pave the way for an integrated, portable, user-friendly, cost-effective, and reliable pathogen detection device. This In Vitro Diagnostic (IVD) device employs the mixing frequency detection technique to identify the presence and concentration of magnetic nanoparticles used as markers in pathogen detection within a microfluidic channel. The detection of these MNPs indicates the presence or absence, as well as the concentration, of the targeted pathogen in a biological sample such as blood, saliva, or urine.

The design of the miniaturized magnetic detection system prototype, including its electrical and magnetic components, as well as the fabrication and installation of the microfluidic microstructure, is detailed in Section 2. Then, in Section 3, three microfluidics structures are proposed, designed, and fabricated. A 3D printing technique is used for mold fabrication of the microfluidics chips; then, the best chip is chosen to enhance the performance of the system and increases the sensing voltage. In Section 4, the fabricated microfluidics structures are tested, and their performances are compared and discussed. Finally, in Section 5, the results and structures are evaluated, and conclusions are provided.

## 2. Miniaturized Magnetic Detection System

Magnetic biosensors operate by measuring magnetic-field strength and frequencies, which are modified by the amount of the analyte within the biological sample under test. Within Lab-On-Chip (LOC) setups, magnetic beads are commonly employed, binding to analytes through antigen–antibody sandwich immunoassays [14]. The objective of magnetic techniques is to identify the magnetic nanoparticles (MNPs) rather than directly detecting the analyte itself.

### 2.1. Mixing Frequency Technique 

**Figure 1:**
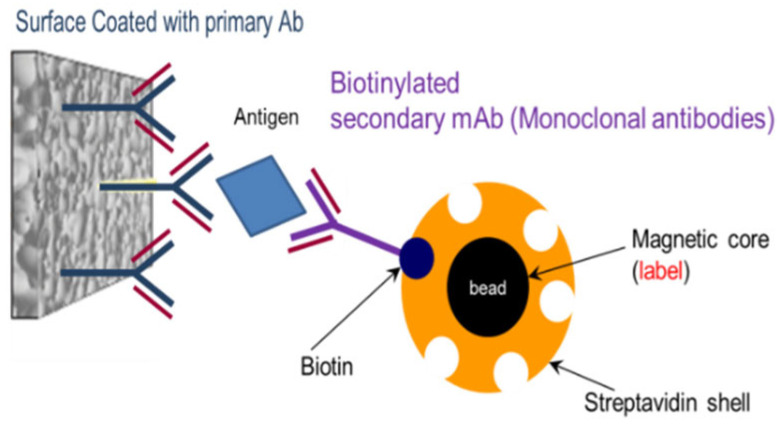
Magnetic detection immunoassay sandwich configuration.

The pathogen detection method is based on the antibody–antigen interaction, which is prepared with a sandwich-like configuration. As illustrated in Figure 1, primary antibodies (Ab) are attached to the surface of the sample holder. In order to detect the target antigens, they should attach to antibodies coated on the surface of the sample holder. Superparamagnetic nanoparticles (SPNs) are coated by streptavidin, which is linked to biotinylated secondary monoclonal antibodies (mAb). During a sequential procedure, different fluids containing the antibodies, antigens, and SPNs are trapped on the surface of the sample holder. The antigen is sandwiched between biotinylated secondary antibodies and primary antibodies coated on the sample holder’s surface. This sandwich configuration of the antigen–antibody complexes facilitates the detection of the superparamagnetic nanoparticles in the device, which can be detected when magnetic fields are applied.

**Figure 2:**
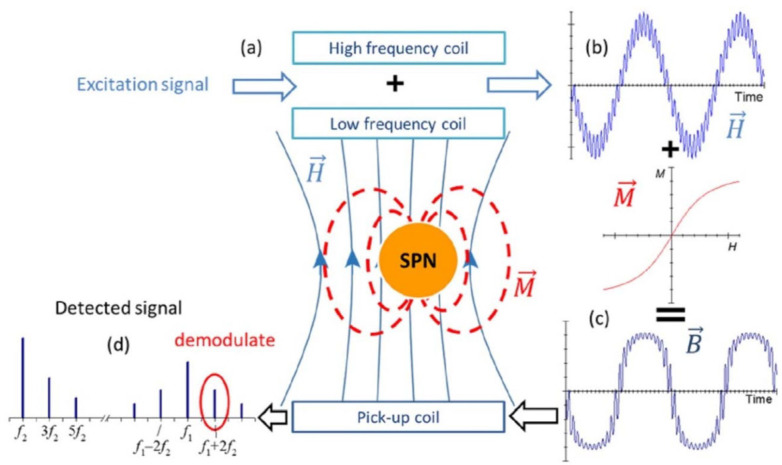
(**a**) The structure of the proposed detection system; (**b**) alternative excitation field; (**c**) resulting magnetic flux density curve; (**d**) Fourier transform of the measured signal. The selected mixing term for the detection of SPN (*f*_1_ + 2*f*_2_) is marked by a red circle [16,17].

Regarding the nonlinearity and absence of hysteresis in the curve of magnetization of SPNs, an innovative method can be applied to detect the concentration of SPNs in the sample. The mixing frequency technique can detect the amount of SPNs or, equivalently, the amount of pathogens trapped in sample holder [15]. The concept involves applying two magnetic fields, each operating at distinct frequencies—a high frequency denoted as *f*_1_ and a low frequency denoted as *f*_2_—to induce magnetic excitations. The detection of the response signal occurs at a frequency characterized by *mf*_1_ *+ nf*_2_, a linear combination of *f*_1_ and *f*_2_ frequencies, where *m* and *n* are integers (Figure 2). The presence of this frequency directly correlates with the nonlinearity observed in the magnetization curve of the SPN, as the low frequency facilitates the SPN’s entry into the nonlinear magnetization region, while the high frequency interrogates this nonlinearity. The coils are used both to generate excitation signals and to detect the response signal. In summary, if there are superparamagnetic nanoparticles within the sample under test, the response signal exhibits mixed terms. Conversely, if the sample lacks SPNs, only the two fundamental frequencies (*f*_1_ and *f*_2_) of the stimulating magnetic fields are detected in the response signal. The magnitude of the measured signal is directly proportional to the quantity of nanoparticles present in the sample. Typically, the *f*_1_ + 2*f*_2_ frequency is employed for measurement purposes, as other mixed terms offer diminished sensitivity for quantitative tests.

### 2.2. Miniaturizing the Coils on PCB

This study involves the development of a prototype for a compact detection device capable of identifying superparamagnetic nanoparticles. The device utilizes planar printed circuit board (PCB) coils that apply the excitation signals and measure the response signal. A microfluidics structure is fabricated by polymer, which keeps the SPNs and is placed between the parallel PCBs. Electrical and instrumentation equipment are used to generate the excitation signals to receive and process the response signal. Specialized flow control tools are used to achieve the desired fluid flow rate in the microfluidic channels. The final target is to implement a portable, rapid, and low-cost system for pathogen detection applications in the protection of biodiversity and ecosystems.

**Figure 3:**
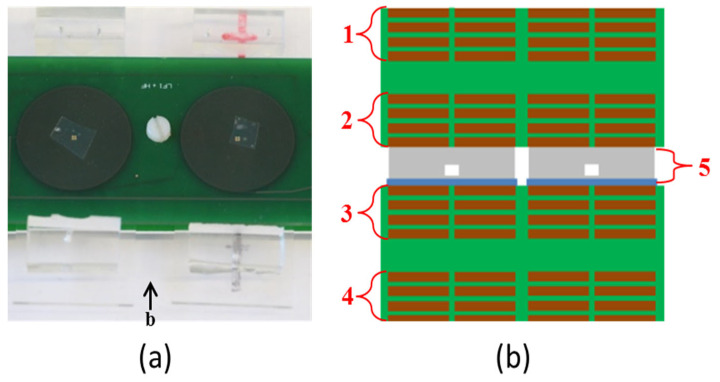
(**a**) PCB and microfluidic pathogen detection device; (**b**) cross section of the coils and layers; 1 and 4 represent 4 layers of low-frequency coil, and 3 and 2 are the detection and high-frequency excitation coils, and 5 is the microfluidics device, respectively.

Planar coils implemented on PCB enable the system to be more compact compared to standard spiral cylindrical coils. A PCB–microfluidic system was fabricated, consisting of three copper planar coils and a polymeric microfluidics structure. All substances consumed in the system are entirely nonmagnetic in order to prevent the production of magnetic, parasitic, or drift signals. Two of the coils generate electromagnetic fields—one at a low frequency and the other at a high frequency—while the third coil functions as the detection coil. The magnetic coils are implemented in two parallel PCB structures with dimensions of 100 × 40 × 1.55 mm^3^. A microfluidic structure that contains the fluids is placed between the PCBs. In this paper we evaluate different shapes and sizes for the microfluidics section, but a typical surface would be 12 × 12 mm^2^, which can hold approximately 14 μL of magnetic nanoparticle suspension. Each coil consists of four layers, with tracks that are 100 μm wide and spaced 100 μm apart. The thickness of each track layer is 35 μm. The emitting coils are fabricated with a radius of 13 mm in 60 turns per layer, while the detection coil has a radius of 10 mm with 46 turns per layer. Since both the excitation and detection coils are built into the same PCB, careful balancing of the specified parameters is crucial for effective magnetization and detection. The distance between the PCBs is adjustable to 2.4 mm or more. The Polydimethylsiloxane (PDMS) microstructure is bonded on 1 mm thick glass, so there is a 1 mm gap between the microfluidic reservoir and the detection coil, which is fabricated in the lower PCB. Furthermore, in order to reduce the effect of environmental magnetic noise on the measured signal, two similar coils with the same number of turns and inductance are fabricated on the same PCB. One of them produces a reference blank signal, and the other produces the measured sample signal. The differential method is applied, and the subtraction of these measurements is analyzed in next steps. In this way, external magnetic noises are reduced. The planar coils are fabricated as shown in Figure 3a,b. In order to find the best configuration, different arrangements of the coils were fabricated and tested. Finally, the best fabricated configuration was chosen, as shown in Figure 3a.

**Figure 4:**
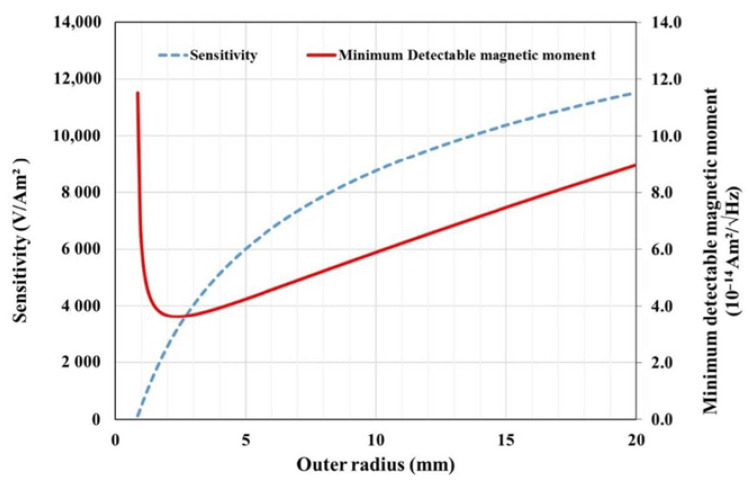
Optimization of the pick-up coil and determination of the external radius of the coil with respect to the sensitivity and minimum detectable moment.

The outer radius of the pick-up coil can be optimized by balancing between the minimum detectable magnetic moment and the sensitivity. Figure 4 illustrates the optimal sensing performance achievable under PCB constraints, such as copper thickness, inter-layer distance, and the smallest practical internal radius. For the selected manufacturer, the copper track section is 35 μm × 100 μm, and the minimum inner radius is 800 μm. Calculations were performed for a four-layer PCB coil. As shown in Figure 4, when the external radius exceeds 3 mm, the minimum detectable magnetic moment increases. It appears that there is a trade-off between sensitivity and the smallest detectable magnetic moment. In this research, for the detection of small magnetic moments while achieving an acceptable value of sensitivity, the selected outer radius is approximately equal to 10 mm.

The dimensions, total number of turns, number of layers, and other specifications of coils on the PCB can be seen in Table 1.

An impedance analyzer measures the impedance of the coils, and a Gaussmeter defines the magnetic field on the surface of the excitation and sensor PCB coils. Direct currents with different voltage values (3, 4, and 6 V) are applied to the coils in order to measure the excitation magnetic fields. The findings are noted in Table 2.

In order to measure the concentration of SPNs in microfluidic structures, a magnetic field is applied by the excitation coils, which are powered by two different current sources at the *f*_1_ and *f*_2_ frequencies. The signal from the detection coil is then amplified and demodulated to produce a response in the form of a DC value. The LF coils are derived by a 48 Vpp, low-frequency, 65 Hz signal, while the HF coils are excited at a high frequency at 40 kHz and 40 Vpp amplitude. Afterward, the signal response is measured by the detection coil and amplified and demodulated using a lock-in amplifier in order to produce the output DC value.

### 2.3. Microfluidics Structure for Immunoassays

In the previous section, we proposed miniaturizing the magnetic coils, including LF, HF, and sensing coils by implementing them on a multilayer PCB as planar coils. In addition, we used microfluidics structures to reduce the reservoir volumes, leading to reductions in magnetic nanoparticles and biomaterial consumption. The volume of the microfluidics structure is much less than that of traditional experimental setups, and it helps to reduce the volume and mass of consumed materials. For the microfluidics structure proposed in this paper, almost 10 µL–20 µL of reagents are consumed, while using the traditional method, more than 200 µL are usually consumed.

**Figure 5:**
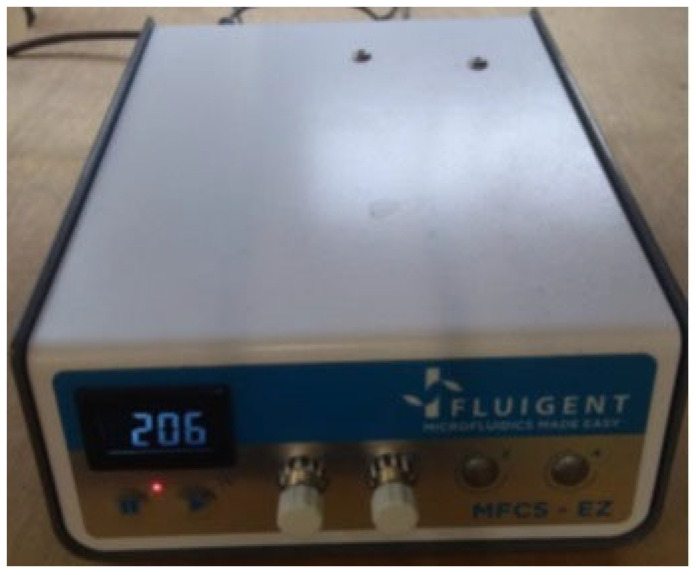
Pressure-controlled pump.

The microfluidic structure serves as the space where magnetic nanoparticles are situated and assessed by the detection system. For suitability in prototyping, PDMS was chosen for the fabrication of the microfluidic structure. In the ultimate device, the microfluidic channel ought to be pre-functionalized prior to the immunoassay, and after each pathogen detection round, it should be thrown away as a disposable microfluidic chip. While cleanable and reusable microfluidic chips are feasible, this approach is complicated, particularly for medical applications. As shown in Figure 5, the flow in the microfluidic structure is derived by a *Fluigent* (founded in 2005, Paris, France) microfluidic pressure-controlled pump. In some papers and applications, the syringe pumps have been utilized, but they are not fully suited for microfluidic channels because they regulate the flow rather than the pressure in the channels. If a blockage occurs, this can lead to a pressure spike that may damage the channel. Pressure-controlled pumps provide a safer and more uniform method for the generation of flow in microfluidic channels.

The pressure-controlled pump is software-controlled, allowing for the application of varying pressures to each outlet. It is connected to a plastic tube containing the fluid, enabling injection into the device. 

**Figure 6:**
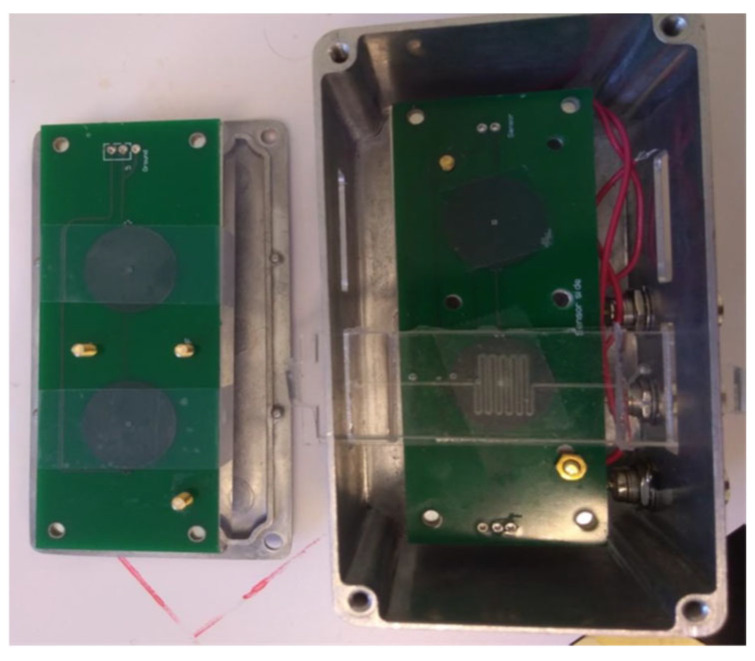
Internal view of the Faraday cage and microfluidics device.

In order to shield the magnetic nanoparticle detection system from electromagnetic interference, a metallic box was designed and manufactured to act as a Faraday cage and protect the boards and system from external noises. Although the Faraday cage marginally enhances the detected signal, conducting measurements proves impractical due to the plastic tubes connected for the transfer of the input and output fluid within the microfluidic device. Due to the friction between the tubes and the metal of the cage when opening or closing, there is a possibility of disconnecting the inlet and outlet tubes of the microfluidic device. Consequently, the Faraday cage underwent redesign and was rebuilt to achieve better integration and more functionality. The final system can be seen in Figure 6.

## 3. Microfluidics Design and Fabrication

In this section, various shapes and structures for microfluidic channels and reservoirs are simulated and fabricated. In the next section, the experimental results are presented and compared. The initial goal was to maximize the volume of the reservoir in the microfluidic part, which is fixed between the magnetic excitation coils and the detection coil. Subsequently, the focus shifted to maximizing the surface-to-volume ratio in order to augment the number of nanoparticles trapped in the microfluidic reservoir for pathogen detection. The sandwich configuration requires a bonding surface, and a larger surface-to-volume ratio enables more MNPs to bind, leading to a stronger detected signal.

Three different reservoir and channel designs are proposed in this paper, namely serpentine-shaped channels, spiral-shaped channels, and microchannels with pillars. All of them were fabricated and tested at Sorbonne University.

### 3.1. Serpentin-Shaped Microfluidics

**Figure 7:**
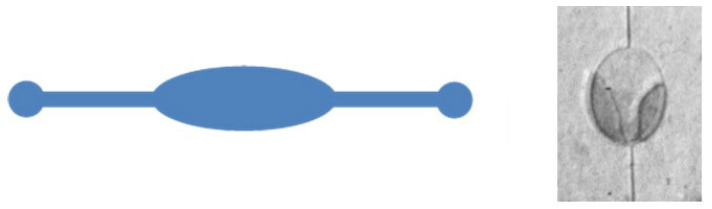
Oval-shaped microfluidics reservoir.

**Figure 8:**
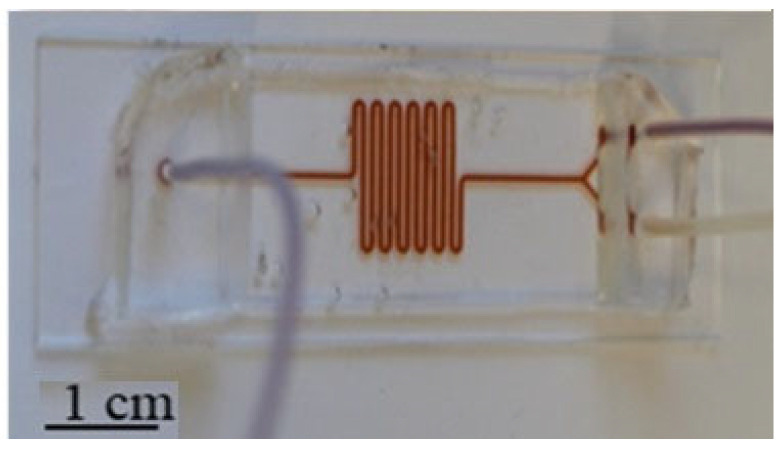
Serpentine microfluidic shape fabricated by PDMS with two inlets and one outlet.

An oval-shaped reservoir, as depicted in Figure 7, was used prior to the serpentine-like microfluidic channel. However, the central zone tended to collapse during experiments because of high surface-tension forces. To address this issue, another stable structure in the form of a serpentine was proposed and implemented, as shown in Figure 8. This serpentine shape maintains the reservoir’s volume uniformity for fluid flow and offers significantly better mechanical stability.

To study the effects of geometrical dimensions on the device’s signal response, two geometrically different serpentine structures were designed, fabricated, and tested. In both designs, the channel width was fixed at 500 µm, while there were two different channel heights, namely 100 µm and 200 µm. In addition, two cases were considered for the serpentine reservoir area, namely 6 × 6 mm^2^ and 12 × 12 mm^2^, as shown in Figure 8. The spacing between the adjacent channels was set to 500 µm to facilitate the fabrication process.

### 3.2. Spiral-Shaped Microfluidics

**Figure 9:**
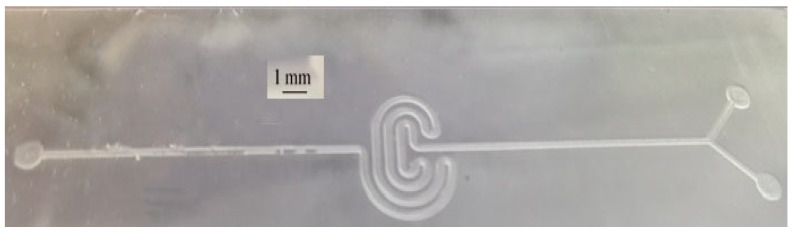
Spiral microfluidic shape fabricated by PDMS.

In spiral geometry, the nanoparticles concentrate in the central zone of PCB planar coils where the strongest magnetic field is created. The channels and reservoir are aligned well with the electromagnetic coil turns. Considering that the magnetic field produced by the LF and HF currents is relatively uniform and strong at the center of the coils, this design enhances the magnetic excitation of SPNs and produces a larger detection signal for a definite number of SPNs. It would generate a larger response signal for the same quantity of SPNs in the microfluidic device. As shown in Figure 9, the channel width and height are 500 µm and 200 µm, respectively.

### 3.3. Pillar-Based Microfluidics Shape

As previously mentioned, the aim of this paper is the design and fabrication of a microfluidic device for magnetic pathogen detection based on the creation of a sandwich immunoassay on the surface of a sample holder. To maximize the number of nanoparticles bound to the surface, efforts were made to increase the surface-to-volume ratio of the reservoir in the microfluidic device. One method to achieve this goal is to create pillars within the reservoir. In this way, it is possible to also reduce the required sample volume for a round of testing, which also enhances the mixing quality of the materials in the device by passing the flow, which improves suspension homogeneity.

**Figure 10:**
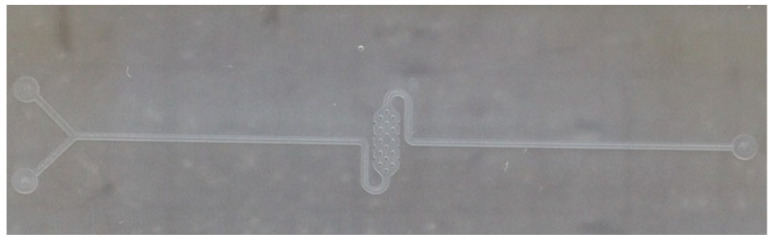
Pillar-based microfluidics structure fabricated by PDMS.

The radius of each pillar is 200 µm, and the dimensions of the reservoir are 2 mm (width) × 6 mm (length). The surface-to-volume ratios in serpentine and spiral reservoirs are very similar and equal to 9000:1 (9000 cm^2^/cm^3^). On the other hand, in the pillar-based reservoir, the surface-to-volume ratio is almost 11,500:1, which is more than in previous cases. The fabricated pillar-based microfluidics structure is shown in Figure 10.

### 3.4. Microfluidics Fabrication Process

**Figure 11:**
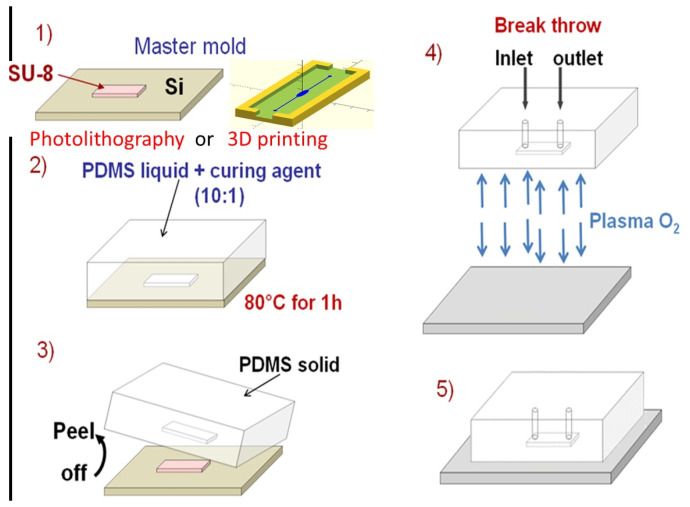
Schematic illustration of the fabrication process.

Various methods, such as photolithography and 3D printing, have been proposed for the fabrication of microfluidic structures using PDMS. These fabrication techniques are illustrated in Figure 11. The quick and simple method involves 3D printing. Initially, a master mold with an acceptable resolution is printed. Then, the liquid monomer and curing agent are poured onto the master mold after degassing the mixture. 

As mentioned above, the dimensions of microfluidics chips, including the channels and reservoirs, are relatively large, ranging between 50 μm and 200 μm. Therefore, the most simple and low-cost fabrication process, i.e., the 3D printing technique, was applied instead of a complex and expensive photolithography process.

## 4. Simulation and Experimental Results

In this section, the results of several simulations and experimental tests are presented. COMSOL Multiphysics version 5.3 was used to simulate the surface concentrations, concentration profiles in fluid flow, fluid velocity profile (including potential dead volume), and reaction kinetics in order to select the most suitable design. The simulation results help us to compare the specifications of different designs rather than replicate an exact behavior, particularly concerning surface reaction kinetics. Modules related to surface reactions, laminar flow, and the transport of diluted species were utilized for this purpose.

### 4.1. Simulation Results

**Figure 12:**
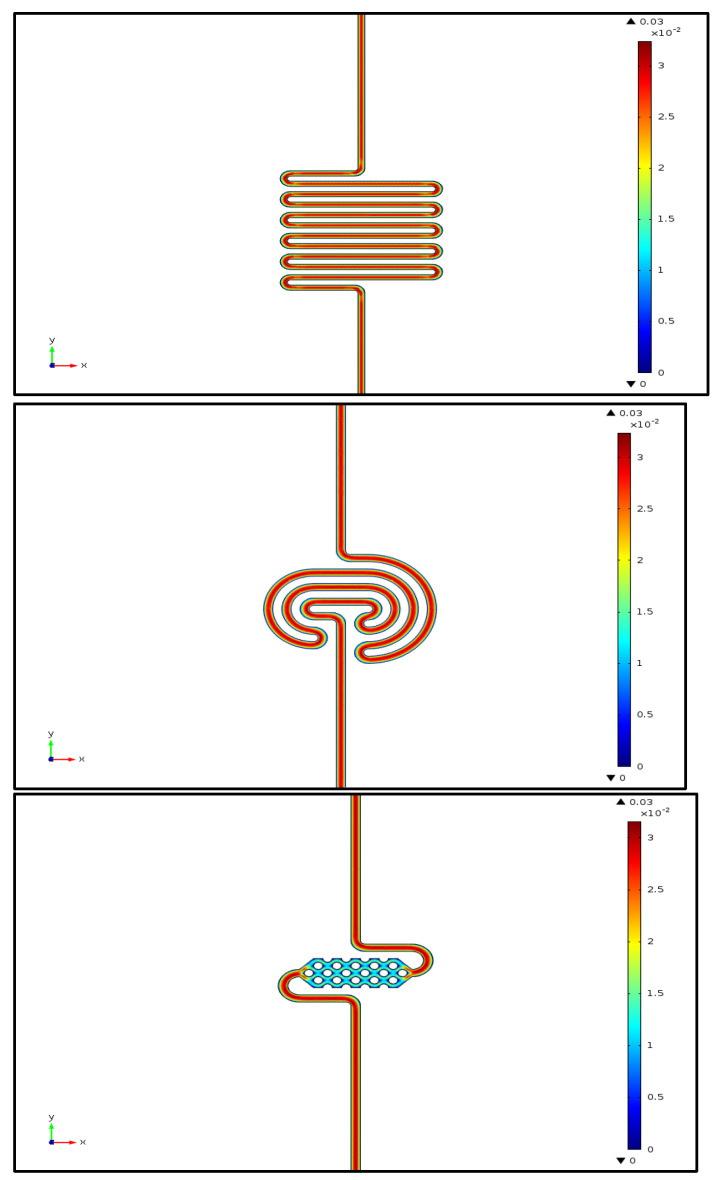
Fluid velocity profile (m/s) under a steady-state condition in different microfluidic reservoirs. The fluid enters from the bottom and comes out at the top of the images. Simulation results of serpentine, spiral, and pillar-based reservoirs.

The simulation results, as shown in Figure 12, indicate that fluid flow in the serpentine and spiral channel shapes remains quite constant. However, when the fluid enters the reservoir containing pillars, the flow rate is reduced by half. This reduction is due to the higher fluidic resistance caused by the presence of the pillars. Despite this, the slower flow rate actually enhances interactions between antibodies and antigens in the central region of the device, making this microfluidic channel design advantageous. However, some dead volumes are observed on the sides of the pillars and in the corners located at the edges of the reservoir. It can be concluded that there would be a lack of homogeneity in the surface species within the reservoir over time, at least until saturation and a steady-state condition are achieved.

**Figure 13:**
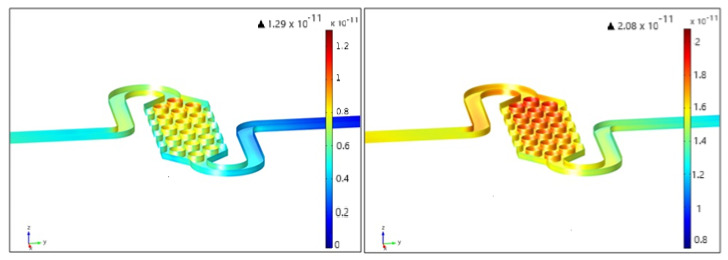
Analyte surface concentration in the pillar geometry after 5 s of flow (**left**) and 10 s of flow (**right**).

COMSOL simulations of antigen surface concentrations were performed for the three different proposed reservoirs at various adsorption rates. Figure 13 illustrates the predicted lack of homogeneity in the immobilization of the analyte on the internal surface of the chip based on the fluid velocity profile. The variation in surface concentrations around the pillars could pose challenges in achieving high sensitivity in the device. This issue might be mitigated by optimizing the flow duration before taking measurements.

**Figure 14:**
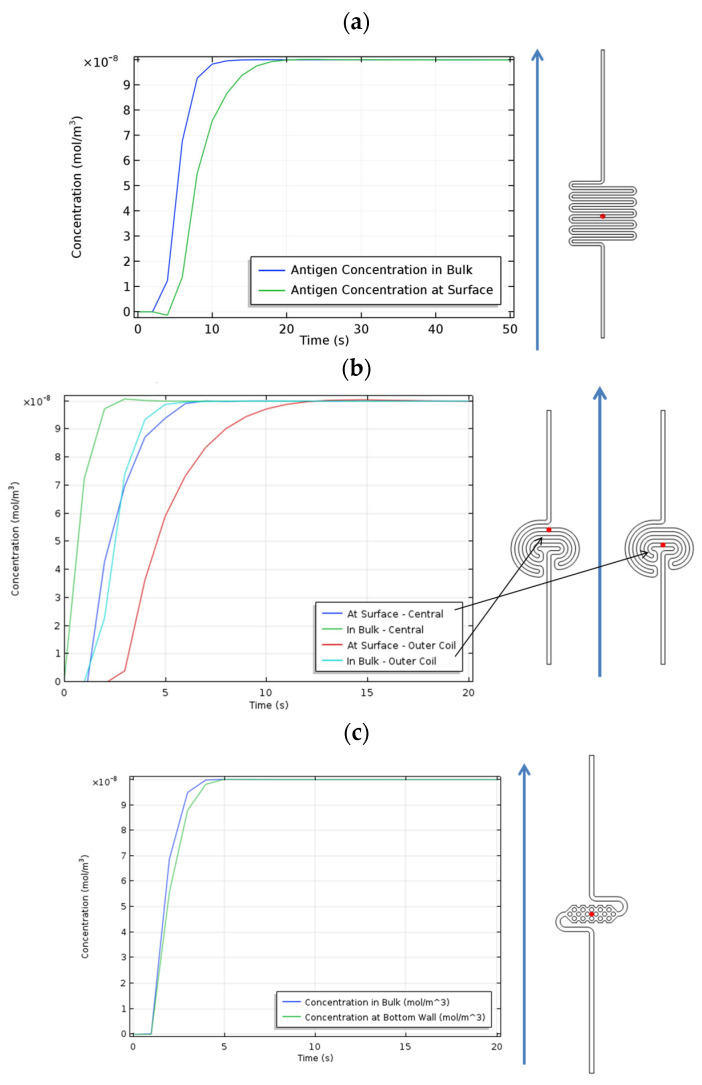
The antigen concentrations in reservoirs versus time in (**a**) serpentine, (**b**) spiral, and (**c**) pillar-based structures. The red dots indicate the location of the points where the concentration is simulated.

The COMSOL simulations help us to more precisely understand the variations in antigen concentration versus time in different microfluidic structures. The antigen concentrations in fluid flow through the microfluidic structures are shown in Figure 14. In all structures, the concentration in bulk increases faster than the surface because the cross-sectional area of the channels is large enough to reduce the influence of surface tension forces, allowing the fluid to flow primarily due to the pressure applied by the pump. Additionally, the time required to reach the maximum concentration is longer for the serpentine structure compared to all other cases. This aligns with the simulated pressure drop across the structures, which indicates that the pressure drop in the serpentine structure is higher than in the other reservoir. The serpentine structure offers greater resistance to fluid flow, resulting in a longer time to reach the maximum concentration. 

### 4.2. Experimental Results

**Figure 15:**
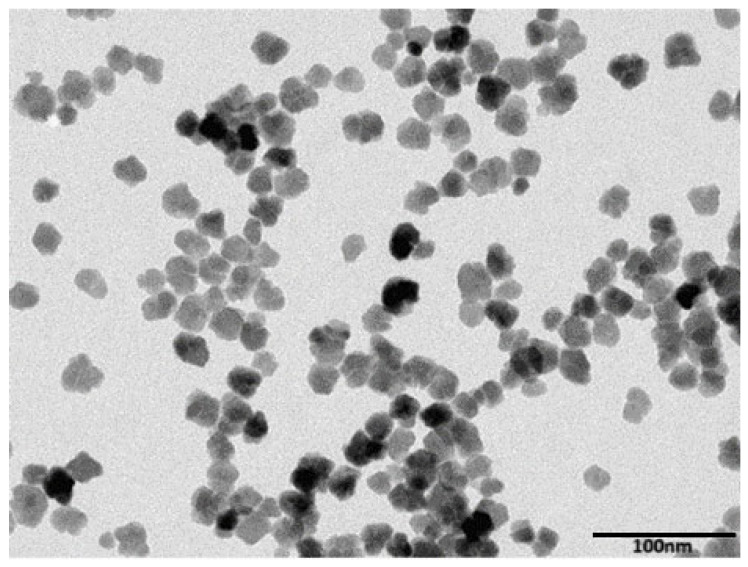
Transmission Electron Microscopy (TEM) image of Magh-20 nm magnetic nanoparticles.

**Figure 16:**
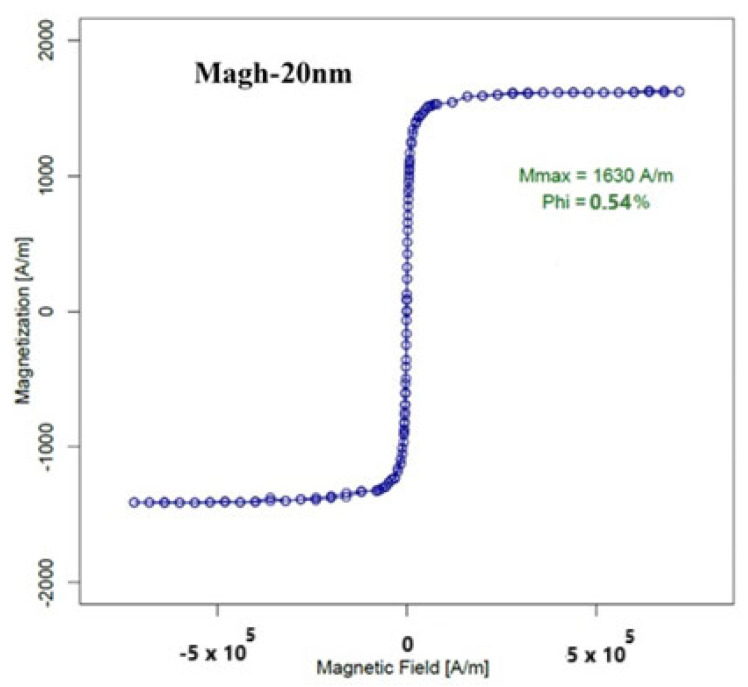
Measured magnetization curve at T = 300 K for Magh-20 nm SPNs.

The magnetic nanoparticles the determine the device’s detection limit are composed of iron oxide (Fe_2_O_3_), also known as maghemite. These nanoparticles, referred to as Magh-20nm, were synthesized and characterized at the PHENIX laboratory. Physical characterizations of these MNPs reveal that (i) the particles have flower-shaped structures with a diameter of 20 nm (Figure 15), and they are stable, even with relatively large diameters, and that (ii) they exhibit superparamagnetic behavior, which means that they are linear before entering the saturation region at 1630 A/m, with no hysteresis in the curve. These characteristics are essential for the application of the frequency mixing technique. The experimental results for magnetization are shown in Figure 16.

The characterizations demonstrate the suitability of these Magh-20nm nanoparticles for the previously outlined frequency mixing technique. The nanoparticles were injected into the microfluidic channel at various concentrations, with the channel surrounded by the PCBs. For stability, flow was halted during magnetic measurements. The LF coils operated at a low frequency of 65 Hz, powered by a 48 Vpp signal, while the HF coils operated at a high frequency of 40 kHz, powered by a 40 Vpp signal. The signal response was amplified by a factor of 200 during demodulation.

For different serpentine structures, the master molds were fabricated by a 3D printer, and the device signal responses were evaluated. In all printed molds, the channel width was adjusted to 500 µm, while the heights of fabricated channels were varied (100 µm and 200 µm). As shown in Figure 8, two different areas of the serpentine reservoir section were assumed, namely 6 × 6 mm^2^ and 12 × 12 mm^2^. The spacing between the channels in the serpentine structure was fixed at 500 µm. The findings are presented in Table 3.

As seen in the table above, altering the channel height directly affects the signal response. For instance, a channel that is two times thinner yields a result approximately two times lower (2 × 2.77 ≈ 5.60). This outcome is logical, since the number of SPNs reacting in the detection zone of the chip, specifically the serpentine reservoir, is directly linked to the reservoir volume.

It can also be found that diminishing the dimensions of the reservoir from 12 × 12 mm^2^ to 6 × 6 mm^2^ does not affect the response signal linearly. This change results in a four-fold reduction in the reservoir’s volume; however, the signal response ratio decreases by less than half $\left(ratio=\frac{5.60}{3.21}=1.74\right)$. This phenomenon is due to the decreasing strength of the excitation magnetic field as one moves away from the central zone of the excitation coils. Consequently, magnetic nanoparticles located in the central zone of the microfluidic chip exhibit a stronger response signal than those situated at the corners. This suggests that we could downsize the microfluidic component with minimal impact on the response signal. Additionally, it implies that enlarging the reservoir size does not proportionally enhance the sensing signal.

Ultimately, it is evident that the spacing between the coils plays a crucial role. A reduction equal to 0.8 mm in height between PCBs from 3.2 to 2.4 mm results in a 1.72 mV increase (almost 44%) in the response signal. Hence, maintaining a close proximity between the coils is essential to uphold the device’s sensitivity.

To evaluate and compare the experimental results of three distinct microfluidic structures, serpentine, spiral, and pillar-based reservoir shapes were all subjected to testing in the magnetic detection device under the same conditions. This included the same SPN size and concentration, magnetic excitation, and sensing parameters. Table 4 presents the results obtained for these three microfluidic structures.

The spiral and pillar-based designs exhibit lower pressure drops due to their shorter channel lengths compared to the serpentine microstructure. The findings indicate a nonlinear relationship between the signal response and the reservoir volume. For instance, despite the serpentine structure having a reservoir volume of 17.28 µL, which is significantly larger than the 1.32 µL volume of the pillar-based structure (almost 13 times more), the measured sensor voltage is approximately 2.5 times higher.

## 5. Conclusions

It is very difficult or impossible to transfer the samples of trees, plants, and other green plants to a professionally equipped laboratory far from the farm or forest to detect biological pathogens that cause infections in plants. Instead, it is very simple, economic, and practical to detect infections in forests, plants, or trees using a portable, low-power, precise, and cheap system, which is the subject of this paper. The proposed detection system has a noticeable impact on protection of the environment, wild plants in forests, and agricultural plants. It is also advantageous to have a portable pathogen detection system in airports, railway stations, and homes to rapidly detect of pathogens in order to prevent pandemics and infections. In this paper, a microfluidics chip in conjunction with electromagnetics planar coils is presented that can detect some kinds of infections by measuring the magnetic response of MNPs conjugated with pathogens. The frequency mixing technique is used to excite MNPs and to measure their responses in order to detect the concentration of MNPs in biological fluid. This method already been applied for pathogen detection in some domains, including agriculture, food, and environmental applications. In this paper, a miniaturized pathogen detection system is proposed that is portable and small in size. The magnetic excitation and detection parts are miniaturized by planar coil technology, and the biological interaction channels and reservoirs are miniaturized using microfluidics technology. Three microfluidic structures were designed, simulated, and fabricated considering different shapes and sizes for microchannels and microreservoirs in order to evaluate their advantages and disadvantages. The simulation results show that the pilar-based structure provides a better detection signal with the same volume of biological fluid in comparison with serpentine and spiral shapes. In addition, measurement results show the importance of the central region of microfluidics device, which, importantly, holds the biological fluid as near as possible to central zone of the planar coils. Simulation and measurement results show that the outer radius of the designed planar coils cannot be increased too much without resulting in a decrease in the minimum detectable magnetic moment. In the other words, increasing the number of turns of the coils enhances sensitivity but degrades the limit of detection.

## Figures and Tables

**Table 1:** Dimensions and specifications of the coils.

	Layers	Rin (mm)	Rout (mm)	Turns/Layer	Total Number of Turns
LF ^1^	4	0.8	10	46	324
	4	3	10	35	
HF ^2^	4	2	9	35	140
Sensor	4	0.8	10	46	184

^1^ Low-Frequency (LF) excitation coil; ^2^ High-Frequency (HF) excitation coil.

**Table 2:** Measured electrical parameters of different coils. The magnetic field is measured for different applied voltages.

	Resistance (Ω)	Inductance (mH)	V = 3 Volt	V = 4 Volt	V = 6 Volt
HF	35	176	697 µT	890 µT	1300 µT
Detection	44	265	708 µT	1010 µT	1450 µT
LF	90	950	581 µT	715 µT	1110 µT

**Table 3:** Dimensions and measured signal in different serpentine microstructures.

Serpentine Microfluidic Reservoirs		Height Between PCBs [mm]	Detected Signal [mV]
Dimensions of Reservoirs [mm × mm]	Height of Channel [µm]		
12 × 12	200	2.4	5.60
12 × 12	200	3.2	3.88
12 × 12	100	2.4	2.77
6 × 6	200	2.4	3.21

**Table 4:** Comparison of the characteristics of serpentine, spiral, and pillar-based structures.

Geometric Design	Serpentine	Spiral	Pillar-Based
Simulated pressure drops (Pa)	900	410	250
Surface-to-volume ratio (m^−1^)	9000	9000	11,500
Reservoir volume (µL)	17.28	8.26	1.32
Test results with MNP suspension (mV)	3.8	2.8	1.5

## Data Availability

The datasets presented in this article are not readily available because the data are part of an ongoing study.
